# Loperamide poisoning resulting in death: a case report and literature review

**DOI:** 10.3389/fphar.2025.1597869

**Published:** 2025-08-22

**Authors:** Bowen Wang, Zhan Zhang, Ke Chen, Zhongyang Du, Tianchen Zhang, Ping Xie

**Affiliations:** ^1^ Department of Cardiovascular Medicine, Gansu Provincial Hospital, Lanzhou, China; ^2^ First Clinical Medical College, Gansu University of Chinese Medicine, Lanzhou, China; ^3^ Department of Cardiovascular Medicine, Wushan County Hospital of Traditional Chinese Medicine, Tianshui, China

**Keywords:** loperamide toxicity, cardiotoxicity, QTc interval prolongation, adolescent poisoning, case report

## Abstract

Loperamide is a medication commonly used to treat acute and chronic diarrhea and is generally considered safe because it poorly crosses the blood-brain barrier at therapeutic doses. However, in recent years, with the abuse and overdose of loperamide, its potential cardiotoxicity and central nervous system depression have increasingly raised concerns. This article reports a case of a 15-year-old male patient who died from poisoning after a single ingestion of 60 mg of loperamide. The patient took a large dose of loperamide 6 h before admission and was found to have respiratory and cardiac arrest 1 h before admission. Despite cardiopulmonary resuscitation and advanced life support treatment, the patient ultimately died from irreversible brain damage. Electrocardiography showed significant QTc interval prolongation, and the plasma loperamide concentration was 50 ng/mL, indicating loperamide’s cardiotoxicity. This article, through this case and a review of the literature, discusses the mechanisms of loperamide’s cardiotoxicity, clinical manifestations, and treatment strategies, aiming to enhance clinicians' awareness of loperamide poisoning.

## Introduction

Loperamide is a long-acting antidiarrheal drug that works by activating peripheral μ-opioid receptors, inhibiting intestinal smooth muscle contraction, reducing bowel motility, and thus prolonging the residence time of food in the small intestine, promoting the absorption of water, electrolytes, and glucose. Because it poorly crosses the blood-brain barrier, loperamide at therapeutic doses is considered safe, with adverse reactions mostly being mild constipation and dizziness. However, in recent years, with the abuse and overdose of loperamide, its potential cardiotoxicity and central nervous system depression have increasingly raised concerns. Especially at high doses or when used in combination with other drugs, loperamide may inhibit cardiac potassium and sodium channels, leading to QTc interval prolongation, QRS interval widening, and even fatal arrhythmias. Although there are reports of loperamide poisoning causing cardiotoxicity and death abroad, similar cases have not been reported in China. This article, through a case of a 15-year-old male patient who died from poisoning after a single ingestion of 60 mg of loperamide, combined with domestic and international literature, discusses the mechanisms of loperamide’s cardiotoxicity, clinical manifestations, and treatment strategies, aims to enhance clinicians' awareness of loperamide poisoning and provide a reference for future clinical practice. This study was approved by the hospital ethics committee, and informed consent was obtained from the patient’s family.

## Case report

The patient was a 15-year-old male with a previously healthy medical history. He had been taking loperamide hydrochloride capsules for recent diarrhea. Six hours before admission, after emotional fluctuations, the patient self-administered 30 capsules of loperamide hydrochloride (60 mg). One hour before admission, his family found him unresponsive and immediately called 120 (emergency services). When paramedics arrived, the patient had no vital signs and immediately began cardiopulmonary resuscitation and transported him to our hospital’s emergency resuscitation room. Upon detailed retrospective review of the medical history, the patient had no congenital long QT syndrome, no family history of inherited cardiac disorders or sudden cardiac death, and had not taken CYP3A4 inhibitors (including ketoconazole and erythromycin), tricyclic antidepressants, or other QT-prolonging agents within 72 h preceding symptom onset.

**FIGURE 1:**
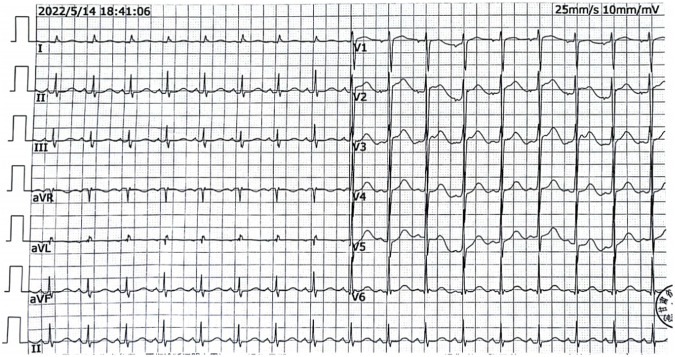
Patient’s electrocardiogram showing QTc interval prolongation to 515 m.

Upon admission, the patient had respiratory and cardiac arrest, dilated and fixed pupils with no light reflex, and a Glasgow Coma Scale score of 3. After 10 min of continuous cardiopulmonary resuscitation and advanced life support, sinus rhythm was restored. Electrocardiography showed Sinus tachycardia (heart rate 112 bpm) with normal axis; QTc interval 515 m; absence of U waves, and no notching or flattening observed in T-wave morphology (Figure 1). Heart rate: 112 beats/min, blood pressure 80/54mmHg.The patient was intubated and placed on mechanical ventilation. Gastric lavage produced approximately 3,000 mL of light yellow fluid, consistent with the color of the ingested medication. After 30 min, the patient’s temperature was 36 °C, pulse was 97 beats/min, respiratory rate was 18 breaths/min (with mechanical ventilation), and blood pressure was 136/96mmHg, maintained with vasoactive agents. The Glasgow Coma Scale score remained 3, and the APACHE II score was 12. A small amount of wet rales was audible in both lungs. The heart rhythm was regular, and no pathological murmurs were heard. The abdomen was soft, the liver and spleen were not palpable, and pathological signs were not elicited. Auxiliary examinations: white blood cell count (WBC) 10.39 × 10^9/L (reference range: 3.5–9.5 × 10^9/L), neutrophil percentage 90.70% (reference range: 40%–75%), alanine aminotransferase (ALT) 60U/L (reference range: 9–50U/L), aspartate aminotransferase (AST) 116U/L (reference range: 15–40U/L), creatine kinase (CK) 18870U/L (reference range: 50–310U/L), creatine kinase isoenzyme (CK-MB) 399U/L (reference range: <25 U/L), lactate dehydrogenase (LDH) 1071.98U/L (reference range: 120–250U/L), cholinesterase (CHE) 6960U/L (4000–12000U/L), potassium level 4.3 mmol/L (reference range 3.5–5.1 mmol/L), magnesium level 0.76 mg/dL (reference range 0.75–1.02 mg/dL), serum creatinine 52umol/L (reference range 57–111 umol/L), troponin I elevated 2.56 ng/mL (reference range <0.4 ng/mL), and the plasma loperamide concentration obtained approximately 8 h after admission was 50 ng/mL (reference therapeutic range 2.0–3.1 ng/mL). Bedside chest X-ray showed no obvious abnormalities. Continuous advanced life support, naloxone (1 mg IV bolus followed by 0.4 mg/h continuous infusion; consciousness remained unresponsive at cumulative 19.2 mg dose), polarizing solution (10% glucose 500 mL + regular insulin 10U + 10% potassium chloride 10 mL + 50% magnesium sulfate 10 mL), and hemoperfusion (HA230 resin perfusion device, 2 units) were administered as symptomatic treatment. After 18 h of resuscitation, the patient did not regain consciousness, had no spontaneous breathing, blood pressure was difficult to maintain, and pupils were dilated and fixed. With no improvement, the family gave up resuscitation, and the patient was pronounced clinically dead.

## Discussion

This case report details a 15-year-old male patient who died from poisoning after a single ingestion of 60 mg of loperamide. The patient presented with respiratory and cardiac arrest upon admission, and electrocardiography showed significant QTc interval prolongation (515 m). The plasma loperamide concentration was 50 ng/mL, indicating loperamide’s cardiotoxicity. Despite intensive resuscitation efforts, the patient succumbed to irreversible brain damage.

Loperamide is a long-acting antidiarrheal drug that works by activating peripheral μ-opioid receptors (MOR), inhibiting intestinal smooth muscle contraction, reducing bowel motility, and prolonging the residence time of food in the small intestine, promoting the absorption of water, electrolytes, and glucose (Wu and Juurlink, 2017; Eggleston et al., 2020). At therapeutic doses, loperamide poorly crosses the blood-brain barrier and is therefore considered safe (Eggleston et al., 2020; Daniulaityte et al., 2013). Loperamide exhibits dose-dependent pharmacokinetics: the elimination half-life (t_1_/_2_) ranges from 9.1 to 14.4 h at therapeutic doses, while overdose conditions prolong it to approximately 34 h (Eggleston et al., 2015). However, with increasing high-dose misuse or abuse, its potential central nervous system depression and cardiotoxicity have increasingly raised concerns. The cardiotoxicity of loperamide and its metabolite N-desmethyl loperamide is mainly mediated by inhibiting cardiac delayed rectifier potassium channels and sodium channels, leading to QTc interval prolongation and QRS interval widening, which can lead to fatal arrhythmias (such as torsades de pointes, TdP) (Vaz et al., 2018; Sheng et al., 2017; Klein et al., 2016; Litovitz et al., 1997; Bhandari et al., 2024). Among these, N-desmethyl loperamide has a 10-fold higher affinity for human Ether-à-go-go-Related Gene (hERG) channels than the parent drug, and the weaker hepatic metabolism in adolescents may lead to the accumulation of this metabolite, exacerbating the risk of QTc prolongation. The occurrence of cardiotoxicity is related to factors such as medication dose, duration of use, young age, and concomitant medication (Klein et al., 2016; Eggleston et al., 2017; Kang et al., 2016).

There are several case reports abroad of loperamide poisoning leading to cardiotoxicity, with patients mostly being adults taking high doses (40–400 mg) and often having a history of opioid abuse. This case differs in that the patient was younger (15 years old) and took a relatively low dose (60 mg) but still developed severe cardiotoxicity and death. This indicates that even at lower doses, loperamide can cause fatal cardiotoxicity in adolescents. This case highlights the need to re-evaluate the safety of loperamide use in adolescents and suggests developing age-specific dosing guidelines.

Treatment of loperamide poisoning is mainly symptomatic and supportive. For patients with respiratory depression, naloxone should be administered promptly to antagonize the opioid-like effects (Litovitz et al., 1997), with an initial dose of 0.4 mg and continuous infusion if necessary. For cardiotoxicity, electrocardiographic monitoring should be performed, electrolyte imbalances (such as sodium, potassium, calcium, and magnesium) should be corrected, and drugs that can cause QT/QTc interval prolongation should be avoided. Treatment of TdP can include magnesium, isoproterenol, cardioversion, and pacing (Litovitz et al., 1997). It should be emphasized that current research suggests intravenous lipid emulsion may mitigate loperamide-induced cardiotoxicity refractory to conventional supportive care, though its efficacy requires further clinical validation (Sohn, 2023). Notably,HA230 resin hemoperfusion accelerates toxin clearance through specific adsorption of lipophilic loperamide molecules. The HA230 resin hemoperfusion accelerates the elimination of lipophilic loperamide through specific adsorption and is recommended to be initiated within 12 h of poisoning to potentially reverse electrophysiological abnormalities and reduce the risk of ventricular arrhythmias. This technique should be performed in conjunction with naloxone antagonism and electrolyte correction. This case reveals that the risk of loperamide cardiotoxicity may be significantly underestimated and that the risk of loperamide cardiotoxicity may be significantly underestimated and that it exhibits higher lethal sensitivity in adolescents. Adolescents may have immature hepatic metabolic enzymes (such as CYP3A4), leading to the accumulation of the active metabolite (N-desmethyl loperamide), which can exacerbate QTc interval prolongation and the risk of TdP through hERG potassium channel inhibition. In clinical practice, strict adherence to dose limits is necessary, and close attention should be paid to dynamic changes in the QTc interval.

## Data Availability

The raw data supporting the conclusions of this article will be made available by the authors, without undue reservation.
