# Supplementary material for: Loperamide poisoning resulting in death: a case report and literature review
Source: Front Pharmacol. 2025 Aug 22;16:1597869. doi: 10.3389/fphar.2025.1597869 (PMC12411950; doi:10.3389/fphar.2025.1597869)
Supplement: Supplementary file 1 [file Supplementaryfile1.docx]

**Loperamide Poisoning Resulting in Death: A Case Report and Literature Review**

**（CARE Checklist）**

### ****1. Title****

**Loperamide Poisoning Resulting in Death: A Case Report**

### ****2. Key Words****

**Loperamide toxicity, Cardiotoxicity, QTc prolongation, Adolescent poisoning, Fatal arrhythmia**

### ****3a. Introduction****

This case highlights the fatal cardiotoxicity of loperamide in a previously healthy adolescent—a demographic underrepresented in existing literature. While loperamide is considered safe at therapeutic doses, this report demonstrates its lethal potential even at a relatively low overdose (60mg) in adolescents, emphasizing age-specific metabolic vulnerabilities and the need for revised clinical guidelines.

### ****3b. Main Symptoms/Clinical Findings****

**Primary Symptoms**: Respiratory and cardiac arrest, unresponsiveness, fixed dilated pupils.

**Key Clinical Findings**:

QTc interval prolongation (515ms), sinus tachycardia.

Elevated cardiac biomarkers (troponin I: 2.56 ng/mL; CK-MB: 399 U/L).

Plasma loperamide concentration: 50 ng/mL (therapeutic range: 2.0-3.1 ng/mL).

### ****3c. Diagnoses, Interventions, Outcomes****

**Diagnosis**: Acute loperamide-induced cardiotoxicity with fatal arrhythmia.

**Interventions**:

Cardiopulmonary resuscitation (CPR), mechanical ventilation, gastric lavage.

Naloxone infusion, hemoperfusion (HA230 resin device), vasoactive agents.

**Outcome**: Death due to irreversible brain damage and refractory cardiac arrest.

### ****3d. Conclusion****

This case underscores the underestimated risk of loperamide cardiotoxicity in adolescents, even at low doses. Clinicians must prioritize dynamic QTc monitoring, early naloxone administration, and age-specific dosing guidelines to mitigate fatal outcomes.

### ****4. Case Uniqueness (with References)****

This report provides novel insights into adolescent-specific loperamide toxicity. Unlike prior cases involving adults with opioid abuse histories 1010, this adolescent exhibited severe cardiotoxicity at 60mg, likely due to immature CYP3A4 metabolism and N-desmethyl loperamide accumulation 8−98−9. The findings challenge current safety assumptions and advocate for stricter pediatric dosing regulations.

### ****5a. Patient Information****

**Age**: 15 years.

**Gender**: Male.

**Medical History**: Previously healthy, no chronic illnesses or substance abuse

### ****5b. Primary Concerns/Symptoms****

Sudden unresponsiveness, respiratory arrest, and cardiac arrest 6 hours after ingesting 60mg loperamide.

### ****5c. Medical/Family History****

No relevant medical, genetic, or psychosocial history.

### ****5d. Past Interventions/Outcomes****

None reported.

### ****6. Physical Examination Findings****

**Neurological**: Glasgow Coma Scale 3, fixed dilated pupils.

**Cardiovascular**: Sinus tachycardia (112 bpm), hypotension (80/54 mmHg).

**Respiratory**: Bilateral wet rales on auscultation.

### ****7. Clinical Timeline****

**6 hours pre-admission**: Ingestion of 60mg loperamide.

**1 hour pre-admission**: Found unresponsive; emergency services activated.

**On admission**: Cardiac/respiratory arrest; CPR initiated.

**Post-resuscitation**: Persistent coma, hemodynamic instability.

**18 hours post-admission**: Death declared.

### ****8a. Diagnostic Testing****

**ECG**: QTc prolongation (515ms), sinus tachycardia.

**Laboratory**: Elevated CK (18,870 U/L), CK-MB (399 U/L), troponin I (2.56 ng/mL).

**Toxicology**: Plasma loperamide concentration 50 ng/mL.

### ****8b. Diagnostic Challenges****

Rapid progression to cardiac arrest limited early diagnostic testing.

Differentiation from other toxicological or metabolic causes required exclusion of alternative toxins.

### ****8c. Final Diagnosis****

**Primary**: Acute loperamide-induced cardiotoxicity with torsades de pointes.

**Differential Diagnoses**: Opioid overdose, electrolyte imbalance, congenital arrhythmia syndromes (ruled out via history and labs).

### ****8d. Prognosis****

**Immediate**: Poor due to prolonged cardiac arrest and anoxic brain injury.

**Long-term**: Fatal outcome confirmed within 18 hours.

### ****9a. Therapeutic Interventions****

**Pharmacologic**: Naloxone (0.4mg initial dose), vasoactive agents.

**Procedural**: CPR, mechanical ventilation, gastric lavage, hemoperfusion.

### ****9b. Intervention Administration****

**Naloxone**: Continuous infusion after initial bolus.

**Hemoperfusion**: HA230 resin device for 2 cycles.

### ****9c. Intervention Changes****

Vasopressors escalated due to refractory hypotension.

Hemoperfusion added to enhance toxin clearance, but delayed initiation limited efficacy.

### ****10a. Outcomes****

**Clinician-Assessed**: No neurological recovery, hemodynamic collapse.

**Patient Outcome**: Death due to irreversible brain damage.

### ****10b. Follow-Up Results****

**Post-resuscitation ECG**: Persistent QTc prolongation.

**Laboratory**: Worsening cardiac enzyme elevation.

### ****10c. Adherence/Tolerability****

Interventions adhered to per protocol; no tolerability issues noted

### ****10d. Adverse Events****

Refractory hypotension, anoxic brain injury, and eventual death.

### ****11a. Strengths/Limitations****

**Strengths**: Comprehensive toxicological analysis, adolescent-specific metabolic insights.

**Limitations**: Single-case design, lack of pre-mortem arrhythmia documentation.

### ****11b. Literature Discussion****

Loperamide inhibits hERG potassium channels, causing QTc prolongation 88. Adolescents are vulnerable due to CYP3A4 immaturity, leading to N-desmethyl loperamide accumulation 99. Similar cases in adults require higher doses (40-400mg) 1010, highlighting this case’s uniqueness.

### ****11c. Scientific Rationale****

Death resulted from hERG channel inhibition-induced TdP arrhythmia, exacerbated by delayed metabolite clearance.

### ****11d. Take-Away Lessons****

Clinicians must recognize loperamide’s cardiotoxic potential in adolescents, enforce strict dosing limits, and implement immediate QTc monitoring and naloxone therapy in overdoses.

### ****12. Patient Perspective****

Not applicable (Patient deceased; family consented to report publication).

### ****13. Informed Consent****

**Yes** – Obtained from the patient’s family. Ethics committee approval documented.
